# Psychosocial distress in cancer patients undergoing radiotherapy: a prospective national cohort of 1042 patients in Germany

**DOI:** 10.1007/s00432-023-04837-5

**Published:** 2023-05-10

**Authors:** Alexander Fabian, Alexander Rühle, Justus Domschikowski, Maike Trommer, Simone Wegen, Jan-Niklas Becker, Georg Wurschi, Simon Boeke, Mathias Sonnhoff, Christoph A. Fink, Lukas Käsmann, Melanie Schneider, Elodie Bockelmann, Martin Treppner, Anja Mehnert-Theuerkauf, David Krug, Nils H. Nicolay, Young DEGRO Group, Alexander Fabian, Alexander Rühle, Justus Domschikowski, Maike Trommer, Simone Wegen, Jan-Niklas Becker, Georg Wurschi, Simon Boeke, Mathias Sonnhoff, Christoph A. Fink, Lukas Käsmann, Melanie Schneider, Elodie Bockelmann, Martin Treppner

**Affiliations:** 1 Department of Radiation Oncology, University Hospital Schleswig-Holstein, Arnold-Heller-Str. 3, 24105 Kiel, Germany; 2 Department of Radiation Oncology, Medical Center, Faculty of Medicine, University of Freiburg, 79106 Freiburg, Germany; 3 Department of Radiation Oncology, Cyberknife and Radiotherapy, Faculty of Medicine and University Hospital Cologne, 50937 Cologne, Germany; 4 Center for Molecular Medicine Cologne, University of Cologne, 50931 Cologne, Germany; 5 Department of Radiotherapy and Special Oncology, Medical School Hannover, 30625 Hannover, Germany; 6 Department of Radiotherapy and Radiation Oncology, Jena University Hospital, 07740 Jena, Germany; 7 Department of Radiation Oncology, University Hospital Tübingen, 72076 Tübingen, Germany; 8 Center for Radiotherapy and Radiation Oncology, 28239 Bremen, Germany; 9 Department of Radiation Oncology, University Hospital Heidelberg, 69120 Heidelberg, Germany; 10 Department of Radiation Oncology, University Hospital, LMU Munich, 81377 Munich, Germany; 11 Comprehensive Pneumology Center Munich (CPC-M), Member of the German Center for Lung Research (DZL), 81377 Munich, Germany; 12 German Cancer Consortium (DKTK), Partner Site Munich, 81377 Munich, Germany; 13 Department of Radiotherapy and Radiation Oncology, Faculty of Medicine and University Hospital Carl Gustav Carus, Technische Universität Dresden, 01307 Dresden, Germany; 14 Department of Radiotherapy and Radiation Oncology, University Hospital Hamburg-Eppendorf, 20251 Hamburg, Germany; 15 Institute of Medical Biometry and Statistics, University Hospital Freiburg, 79106 Freiburg, Germany; 16 Department of Medical Psychology and Medical Sociology, University Medical Center Leipzig, 04103 Leipzig, Germany; 17 Department of Radiotherapy and Radiation Oncology, University Hospital Leipzig, 04103 Leipzig, Germany; 18 Cancer Center Central Germany, Partner Site Leipzig, 04103 Leipzig, Germany

**Keywords:** Oncology, Radiotherapy, Psychosocial distress, Quality of life, Supportive care

## Abstract

**Purpose:**

Psychosocial distress is common among cancer patients in general, but those undergoing radiotherapy may face specific challenges. Therefore, we investigated the prevalence and risk factors for distress in a large national cohort.

**Methods:**

We performed a secondary analysis of a multicenter prospective cross-sectional study which surveyed cancer patients at the end of a course of radiotherapy using a patient-reported questionnaire. Distress was measured with the distress thermometer (DT), using a cut-off of ≥ 5 points for clinically significant distress. Univariate analyses and multivariate multiple regression were used to assess associations of distress with patient characteristics. A two-sided p-value < 0.05 was considered statistically significant.

**Results:**

Out of 2341 potentially eligible patients, 1075 participated in the study, of which 1042 completed the DT. The median age was 65 years and 49% (511/1042) of patients were female. The mean DT score was 5.2 (SD = 2.6). Clinically significant distress was reported by 63% (766/1042) of patients. Of the patient characteristics that were significantly associated with distress in the univariate analysis, a lower level of education, a higher degree of income loss, lower global quality of life, and a longer duration of radiotherapy in days remained significantly associated with higher distress in the multivariate analysis. Yet effect sizes of these associations were small.

**Conclusion:**

Nearly two in three cancer patients undergoing radiotherapy reported clinically significant distress in a large multicenter cohort. While screening and interventions to reduce distress should be maintained and promoted, the identified risk factors may help to raise awareness in clinical practice.

**Trial Registry identifier:**

DRKS: German Clinical Trial Registry identifier: DRKS00028784.

**Supplementary Information:**

The online version contains supplementary material available at 10.1007/s00432-023-04837-5.

## Introduction

People living with cancer face multiple challenges. On the one hand, a diagnosis of cancer itself or cancer-associated symptoms may negatively impact wellbeing and emotional health. On the other hand, wellbeing and emotional health may also be threatened by cancer-directed therapy e.g., due to fear or presence of side effects. In this context, psychosocial distress (hereinafter referred to as “distress”) is a commonly used multidimensional concept. Distress is defined as “a multifactorial unpleasant experience of a psychological (…), social, spiritual, and/or physical nature that may interfere with the ability to cope effectively with cancer, its physical symptoms, and its treatment. (…)” (Riba et al. 2019). Therefore, screening of distress in cancer patients is recommended and considered standard of high quality cancer care in order to offer support (Riba et al. 2019; Donovan et al. 2020; Leitlinienprogramm Onkologie (Deutsche Krebsgesellschaft, Deutsche Krebshilfe, AWMF) 2022). In fact, distress may arise to varying degrees in cancer patients. Studies have suggested that up to 50% of cancer patients are affected by clinically significant distress (Mehnert et al. 2018; Singer et al. 2019; Wittwer et al. 2022). Risk factors for distress have been described in different cancer patient cohorts and include younger age, female sex or specific cancer diagnoses such as pancreatic cancer (Carlson et al. 2019). However, a more granular view on distress in cancer patients is warranted, as cancer patients could be affected by distress differently depending, for example, on the treatment modality.

Radiotherapy is a key modality in the treatment of cancer. Approximately 50% of all cancer patients in Europe receive at least on course of radiotherapy which may range from one fraction to a course of several weeks (Lievens et al. 2020). Few single center studies with limited sample size have evaluated distress in cancer patients undergoing radiotherapy (Delikanli et al. 2022). In fact, these studies suggest that clinically significant distress may be present in approximately one third of radiotherapy patients (Hess et al. 2015). Furthermore, the presence of distress was associated with worse outcomes ranging from more frequent hospital admissions and missed radiotherapy fractions to lower overall survival (Habboush et al. 2017; Anderson et al. 2019). Since the implementation of distress screening, however, we lack a contemporary and large scaled overview on the prevalence and risk factors for distress in cancer patients undergoing radiotherapy. Therefore, the current analysis aimed at providing a national overview of distress in radiotherapy patients and defining vulnerable subgroups in need of additional supportive measures. Prevalence and associated factors for distress were assessed in a large prospective multicenter survey of radiotherapy patients treated in Germany.

## Materials and methods

This is a post-hoc secondary analysis of a study which had the primary objective to assess financial toxicity in cancer patients undergoing radiotherapy. Study results on financial toxicity have been published previously (Fabian et al. 2023). The present analysis focusses on distress in this cohort.

### Study design and setting

We conducted a prospective, multicenter, cross-sectional study as previously described (Fabian et al. 2022) (Supplementary Document 1). In brief, 11 German study centers recruited eligible patients for an anonymous survey during a period of 60 consecutive days from June 2022 until August 2022. Eligible cancer patients ≥ 18 years had completed a radiotherapy course (end of treatment ± 2 days), were able to understand the questionnaire, provided informed consent, and had not previously participated in this study. This study was carried out in accordance with the principles of the Declaration of Helsinki (2013). Each participating center received Ethics committee approval prior to enrolling patients. We respected the STROBE guideline and CONSORT-PRO extension guideline for reporting the study (Elm et al. 2007; Calvert et al. 2013).

### Questionnaire and variables

Details regarding the questionnaire and collected variables have been presented previously (Supplementary Document 2) (Fabian et al. 2023). In brief, the questionnaire was patient-reported, paper-based, and pilot-tested on eligible voluntary patients. The questionnaire included socio-demographic data, data related to the cancer disease and therapy, employment and financial issues, patient satisfaction, subjective financial distress per question 28 of the EORTC QLQ-C30 questionnaire and global health status/quality of life per question 29 and 30 of the EORTC QLQ-C30 questionnaire (Aaronson et al. 1993). Distress was rated on the validated German version of the NCCN distress thermometer; a valid, reliable, and widely used screening measure (Mehnert et al. 2006). The distress thermometer contains a single‐item visual analogue scale ranging from 0 (“no distress”) to 10 (“extreme distress”) to quantify the global level of distress experienced in the past week including the current day. We used a score of ≥ 5 points as cut-off for clinically significant distress based on previous national literature although we recognize that other cut-offs (e.g. ≥ 4 points) have been proposed in other countries (Mehnert et al. 2006; Luutonen et al. 2011; Hess et al. 2015).

### Statistical analysis

Sample size calculation was based on the primary objective of the study (Fabian et al. 2023). Analyses presented here are exploratory. Descriptive statistics were used to display the study cohort. To explore univariate associations of distress and covariables, one-way ANOVA, Spearman correlation, or Pearson correlation were used in dependence of the scale of the respective covariable. In case of significant results of a one-way ANOVA test, Tukey’s post-hoc testing was used. Missing data were excluded in a pairwise fashion. To explore multivariate associations of distress and covariables, we used a multiple regression model. We did not adjust for multiple testing in light of the exploratory design of our analyses (Bender and Lange 2001). A two-sided p-value < 0.05 was considered statistically significant. The software JASP v0.16.4 (JASP Team [2022], Amsterdam, the Netherlands) was used for statistical analyses.

## Results

### Patient characteristics

**Fig. 1:**
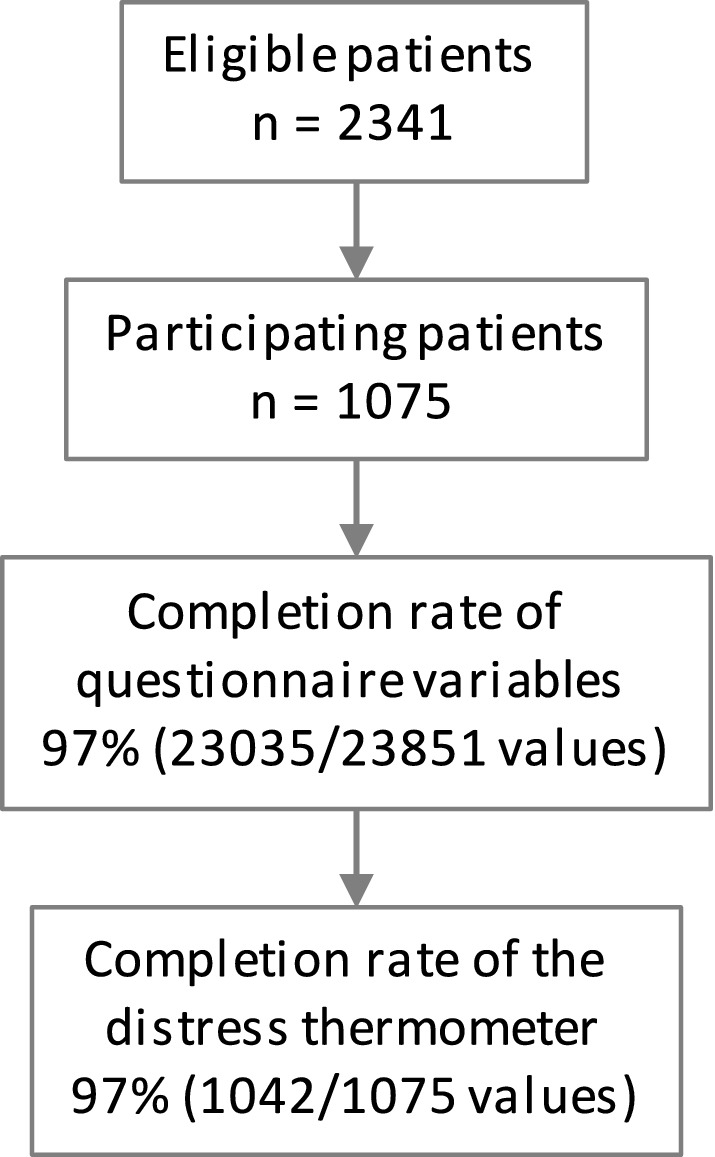
Study flow diagram

The study recruited 1075 of 2341 eligible patients resulting in a participation rate of 46% as previously described (Fig. 1) (Fabian et al. 2023). Of the 1075 participating patients, 97% (1042/1075) completed the distress thermometer. Among those patients answering the distress thermometer, 49% were female and the median age was 65 years (IQR 57–74) (Table 1). The most common tumor entities were breast cancer in 26% (269/1042), prostate cancer in 19% (194/1042), and lung cancer in 10% (102/1042) of patients. The mean duration of radiotherapy was 23 days ± 13. At least some degree of additional direct monetary costs due to radiotherapy was reported by 65% (671/1042) of patients (Supplementary Table 1). At least some degree of loss of income due to radiotherapy was reported by 27% (287/1042) of patients (Supplementary Table 1).

**Table 1:** Characteristics of patients answering the distress thermometer (n = 1042)

Sex	
Male:female	51%:49% (530:511)
Age	Median: 65; IQR: 57–74
Partnership status	
Lives alone	27% (284)
Lives with partner	72% (751)
Education level	
< 10 years of school	31% (320)
10 years of school	35% (365)
> 10 years of school	32% (338)
Health insurance	
Public health insurance	80% (829)
Private health insurance	19% (202)
Employment status	
Employed	29% (300)
Self-employed	5% (58)
Unemployed	8% (86)
Retired	56% (580)
Net household income	
< 1.300 €	19% (202)
1.301–1.700 €	16% (166)
1.701–2.600 €	21% (224)
2.601–3.600 €	15% (161)
3.601–5.000 €	13% (133)
> 5.000 €	6% (58)
Tumor entity	
Breast cancer	26% (269)
Prostate cancer	19% (194)
Lung cancer	10% (102)
Brain tumor (primary or secondary)	7% (76)
Head and neck cancer	7% (72)
Gynecological cancer	4% (38)
Rectal cancer	3% (32)
Other	23% (236)
Duration of radiotherapy	
In days	Mean: 23; SD: 13
Concomitant chemotherapy	
Yes	26% (268)
No	73% (764)
Hospitalized during radiotherapy	
Yes	21% (218)
No	78% (808)
Global health status/QoL	
Per EORTC QLQ-C30	Mean: 55; SD: 22

Absolute numbers are given in brackets. Numbers may not add up to 100% due to rounding error or missing values

*IQR* interquartile range, *QoL* quality of life, *SD* standard deviation

### Prevalence of distress in radiotherapy patients

**Fig. 2:**
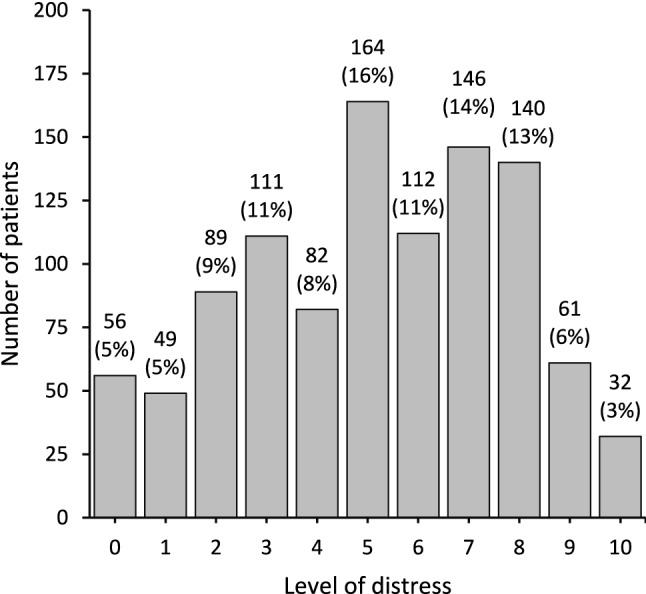
Distribution of distress among cancer patients undergoing radiotherapy (n=1042). Absolute numbers are displayed and percentages are given in brackets

The mean value of distress reported by the patients was 5.2 (SD = 2.6). Distress appeared normally distributed across response categories (Fig. 2). Clinically significant distress was present in 63% (766/1042) of patients.

### Associations of distress with patient characteristics

We conducted univariate analyses to explore potential associations of distress and covariables arising from the questionnaire. Public health insurance, tumor entity, concomitant chemotherapy, and hospitalization were significantly associated with higher distress among categorical covariables per one-way ANOVA (Table 2). Tukey’s post-hoc testing revealed head and neck cancer as the tumor entity significantly associated with higher distress compared to other tumor entities (p < 0.001).

**Table 2:** Association of distress and categorical independent variables per one-way ANOVA (n=1042)

Variable	N	Mean	SD	p
Sex				0.599
Female	511	5.1	2.6	
Male	530	5.4	2.6	
Patient lives				0.818
Alone	284	5.2	2.6	
With partner	751	5.1	2.7	
Health insurance				**0.044**
Public	829	5.3	2.6	
Private	202	4.9	2.8	
Employment status				0.170
Employed	300	5.2	2.7	
Self-employed	58	5.1	2.6	
Unemployed	86	5.8	2.5	
Retired	580	5.1	2.6	
Tumor entity				** < 0.001**
Breast cancer	269	4.8	2.6	
Prostate cancer	194	4.8	2.7	
Lung cancer	102	5.1	2.9	
Brain tumor (primary or secondary)	76	5.5	2.4	
Head and neck cancer	72	6.5	2.1	
Gynecological cancer	38	5.3	2.4	
Rectal cancer	32	5.9	2.3	
Other	216	5.3	2.7	
Concomitant chemotherapy				**0.009**
Yes	268	5.5	2.6	
No	764	5.0	2.7	
Hospitalized during radiotherapy				**0.001**
Yes	218	5.7	2.6	
No	808	5.1	2.6	

Statistically significant *p*-values < 0.05 are displayed in bold

*SD* standard deviation

All available ordinal covariables were significantly associated with higher distress. This included lower net household income (Spearman rho, − 0.088 [95% CI, − 0.151 to − 0.024]; p = 0.007), lower education level (Spearman rho, − 0.083 [95% CI, − 0.144 to − 0.022]; p = 0.008), higher degree of additional costs (Spearman rho, 0.138 [95% CI, 0.073–0.202]; p < 0.001), and higher degree of loss of income (Spearman rho, 0.135 [95% CI, 0.073–0.195]; p < 0.001). Among continuous covariables, age was not associated with distress (Pearson’s r, − 0.036 [95% CI, − 0.097–0.025]; p = 0.224). However, longer duration of radiotherapy in days (Pearson’s r, 0.096 [95% CI, 0.035–0.157]; p = 0.002) and lower global health status/quality of life (Pearson’s r, − 0.550 [95% CI, − 0.591 to − 0.505]; p < 0.001) were significantly associated with higher distress.

We performed a multivariate analysis using a multiple regression model adjusting for age and gender. Distress was used as dependent variable. All statistically significant covariables elaborated in *3.3* were simultaneously entered into the model as independent variables. The multiple regression model statistically significantly predicted distress, F(10,710) = 41.4, p < 0.001, adj. R^2^ = 0.36 (Supplementary Table 2). Lower education level, higher degree of loss of income, longer duration of radiotherapy in days, and lower global health status/ quality of life remained significantly associated with higher distress (Table 3).

**Table 3:** Predictors of distress in patients undergoing radiotherapy per multiple regression (n=1042)

Independent variables	Dependent variable: Distress			
	B	Lower 95% CI	Upper 95% CI	*p*
(Constant)*	9.935	8.533	11.337	** < 0.001**
Health insurance (public)	− 0.115	− 0.548	0.318	0.603
Tumor entity (Head and Neck cancer)	0.355	− 0.277	0.987	0.271
Concomitant chemotherapy (yes)	0.138	− 0.259	0.536	0.495
Hospitalized (yes)	− 0.069	− 0.482	0.344	0.742
Net household income	− 0.106	− 0.227	0.014	0.084
Education level	− 0.243	− 0.451	− 0.036	**0.022**
Degree of additional costs	0.017	− 0.134	0.168	0.822
Degree of loss of income	0.167	0.029	0.305	**0.018**
Duration of radiotherapy (days)	0.021	0.008	0.034	**0.002**
Global health status/quality of life	− 0.066	− 0.074	− 0.059	** < 0.001**

Statistically significant p-values < 0.05 are displayed in bold font

*Model adjusted for age and sex

## Discussion

In this secondary analysis of a cross-sectional prospective study, we have found a high prevalence of distress among cancer patients undergoing radiotherapy. Several patient characteristics were associated with increased levels of distress, among them education level, loss of income, quality of life, and duration of radiotherapy.

The prevalence of clinically significant distress (≥ 5 points) was 63% in our cohort. A previous German cross-sectional study included over 4.000 cancer patients with various entities as well as treatment settings and reported a prevalence of clinically significant distress of 52% (Mehnert et al. 2018). A further German study evaluated 32 patients with localized breast cancer and 67 patients with brain metastases undergoing radiotherapy. The authors reported 66% and 70% of clinically significant distress, respectively (Cordes et al. 2014). Another study evaluated 311 patients awaiting radiotherapy for gynecological cancer in Australia. The authors reported a prevalence of clinically significant distress of 31% (Gough et al. 2022). Lastly, an Indian cross-sectional study of 600 head and neck cancer patients found clinically significant distress in 56% of patients using a cut-off of 4 points on the distress thermometer (Lewis et al. 2021). In light of these previous studies, the prevalence of distress compares relatively high in our cohort. A possible explanation could be the timing of our survey at the end of a course of radiotherapy. Our data suggest that psychological distress is increased at the end of treatment, e.g. due to the anticipated lack of closer medical care and the initial phase of waiting and hoping for successful treatment outcomes. Possibly, toxicity-related symptoms could have also led to a higher prevalence of distress. The presence of symptoms and perceived problems has previously been associated with higher levels of distress (Mehnert et al. 2018). However, multiple smaller studies that have evaluated distress longitudinally over the course of radiotherapy have consistently shown stable or reduced levels of distress at the end of a radiotherapy course (Hernández Blázquez and Cruzado 2016; Westhoff et al. 2017; Halkett et al. 2018; Delikanli et al. 2022). This makes the timing of our survey and potential toxicity-related symptoms as reason for the relatively high prevalence of distress unlikely. Another possible explanation could be the setting of our study. Its primary objective was to assess financial toxicity. It is possible that participants were influenced by the focus on financial toxicity when answering the question on distress. Financial toxicity has indeed been shown to correlate positively with distress (Fabian et al. 2023). Yet the fact that financial toxicity was associated with a distinct set of risk factors compared to those correlating with distress in the present analysis, makes this explanation as the primordial reason for the observed high levels of distress less likely (Fabian et al. 2023).

Lower education level, higher degree of loss of income, lower global quality of life, and longer duration of radiotherapy were factors significantly associated with higher distress in the multivariate analysis, albeit at low effect sizes. Some of these associations have been previously described in different settings. The Indian study of head and neck cancer patients undergoing radiotherapy reported a significant association of lower socioeconomic status and higher distress in a multivariate analysis (Lewis et al. 2021). The observed association of lower socioeconomic status with distress could also encompass lower education levels and loss of income, although this analogy should be taken cautiously due to expectable cross-cultural differences. Furthermore, previous studies have noted a negative correlation of quality of life or wellbeing with distress. This includes a secondary analysis of the Dutch Bone Metastasis Study, the analysis of gynecological cancer patients awaiting radiotherapy, and an analysis of lung cancer patients treated with systemic therapy (Westhoff et al. 2017; Geerse et al. 2019; Gough et al. 2022). Intriguingly, the association of the duration of radiotherapy and distress has, to our knowledge, not been described before in a multivariate analysis. Although unrecorded confounders may be at play, this finding is of interest in the light of readily available hypofractionated and accelerated radiotherapy regimens for various indications. These treatment courses use higher doses per fraction and are delivered in less fractions compared to classical normofractionated regimens. Examples for these indications are, among others, adjuvant radiotherapy for breast cancer or definitive radiotherapy for prostate cancer (Hickey et al. 2019; Krug et al. 2021). Therefore, the implementation of shortened regimens should be fostered where feasible given potential benefits regarding distress of patients.

Literature suggests that programs for screening of distress could be improved, for example concerning screening implementation or referral strategies (Götz et al. 2019; Donovan et al. 2020; Zimmermann et al. 2022). Already to date, there is high level randomized evidence of beneficial effects of interventions against clinically significant distress. These interventions include, among others, web-based counseling or stepped care approaches and have been tested in various setting (Krebber et al. 2016; Urech et al. 2018; Mundle et al. 2021). In the light of these effective interventions, the awareness and need for screening of distress should be stressed even more. Accordingly, guidelines recommend screening for distress in every cancer patient and to repeat screening in case of evolving circumstances (Riba et al. 2019; Leitlinienprogramm Onkologie (Deutsche Krebsgesellschaft, Deutsche Krebshilfe, AWMF) 2022). Clinically significant levels of distress, for example ≥ 5 points on the distress thermometer, should trigger a clinical encounter to determine needs for psychological, social or medical support (Leitlinienprogramm Onkologie (Deutsche Krebsgesellschaft, Deutsche Krebshilfe, AWMF) 2022).

Although our prospective study offers a large, contemporary, and representative cohort of cancer patients undergoing radiotherapy, there are limitations to our analyses. First, all analyses presented here are secondary post-hoc analyses and should be regarded as hypothesis-generating. Moreover, the effect size of statistically significant associations of patient characteristics and distress was low overall. Second, although the study employed the validated distress thermometer, it did not include the companion symptom and problem list which could have offered more detail (Mehnert et al. 2006). Third, it was not feasible to enrich the data set with additional information from medical records due to the anonymous nature of the survey, which was chosen to increase the participation rate. Lastly, our sample could be subject to a bias in the sense that non-responders were less emotionally distressed and our study may therefore overestimate distress.

## Conclusion

In conclusion, our secondary analysis of a large prospective cohort of cancer patients undergoing radiotherapy has shown high rates of distress. Nearly two in three patients showed clinically significantly distress. Lower education level, higher degree of loss of income, lower global quality of life, and longer duration of radiotherapy were weakly associated with higher distress in a multivariate model. Screening for distress should be maintained and promoted to allow for supportive measures in patients undergoing radiotherapy with clinically significant distress.

## Supplementary Information

Below is the link to the electronic supplementary material.

Supplementary file1 (PDF 194 KB)

Supplementary file2 (PDF 125 KB)

Supplementary file3 (PDF 55 KB)

Supplementary file4 (PDF 65 KB)

## Data Availability

Raw data of this analysis are available from the corresponding author upon reasonable request.
