# Supplementary material for: Psychosocial distress in cancer patients undergoing radiotherapy: a prospective national cohort of 1042 patients in Germany
Source: J Cancer Res Clin Oncol. 2023 May 10;149(11):9017–24. doi: 10.1007/s00432-023-04837-5 (PMC10374761; doi:10.1007/s00432-023-04837-5)
Supplement: Supplementary file 1 — Supplementary file1 (PDF 194 KB) [file 432_2023_4837_MOESM1_ESM.pdf]

Study synopsis

**Title**	**Financial Toxicity in Cancer Patients Treated with Radiotherapy – a jDEGRO Confirmatory Cross-Sectional Study**
Version	v1.0 21.03.2022
Study coordinators	Dr. med. Alexander Fabian [1 2]<br>alexander.fabian@uksh.de; alexander.fabian@uniklinik-freiburg.de<br>Vice coordinators:<br>Dr. med. Alexander Rühle [2], alexander.ruehle@uniklinik-freiburg.de<br>Justus Domschikowski [1], justus.domschikowski@uksh.de<br>[1] Klinik für Strahlentherapie, UKSH Campus Kiel<br>Arnold-Heller-Str. 3, 24105 Kiel<br>[2] Klinik für Strahlenheilkunde, Universitätsklinikum Freiburg<br>Robert-Koch-Straße 3, 79016 Freiburg
Study design	Multicenter, non-interventional, confirmatory, cross-sectional study (anonymous questionnaire)
Study population	<u>Inclusion criteria:</u><br>• Cancer patient about to complete (+/- 2d) a course of radiotherapy<br>• Ability to understand and complete the questionnaire<br>• > 18 years<br>• Patient gave informed consent<br><u>Exclusion criterion:</u><br>• Patient has already participated in the study for another course of radiotherapy
Patient number	At least 504 patients based on sample size calculation
Research questions	<u>Primary:</u><br>• Confirmation of the prevalence of financial toxicity<br>• Confirmation of risk factors for financial toxicity<br><u>Secondary:</u><br>• Exploration of additional variables as risk factors for financial toxicity (e.g. distress, duration of radiotherapy)<br>• Association of financial toxicity and patient satisfaction
Statistics	Confirmatory for primary, and exploratory for secondary research questions
Data protection	Anonymous data acquisition
Time frame	• Data acquisition for a 60-day period from May or June 2022<br>• Data analysis until October 2022
Funding	none

Financial Toxicity in Cancer Patients Treated with Radiotherapy – a jDEGRO Confirmatory Cross-Sectional Study

A. Fabian[1 2#], A. Ruehle[2], J. Domschikowski[1], S. Boeke[3], L. Käsmann[4], M. Trommer[5], S. Wegen[5], G. Wurschi[6], D. Krug[1], N. H. Nicolay[2]

[1]Department of Radiation Oncology, University Hospital Schleswig-Holstein, 24105 Kiel, Germany

[2]Department of Radiation Oncology, University Hospital Freiburg, 79106 Freiburg, Germany

[3]Department of Radiation Oncology, University Hospital Tübingen, Germany.

[4]LMU University Hospital, Department of Radiotherapy and Radiation Oncology, Munich

[5]University Hospital Cologne, Clinic and Policlinic for Radiation Oncology, Cyberknife and Radiotherapy, Cologne

[6]University Medical Center Jena, Department of Radiation Oncology, Jena, Germany

# Correspondence address: Alexander Fabian MD, MBA, Department of Radiation Oncology, University Hospital Freiburg, Robert-Koch-Str. 3, 79106 Freiburg, Germany, // Department of Radiation Oncology, University Hospital Schleswig-Holstein, Arnold-Heller-Str. 3, 24105 Kiel, Germany // alexander.fabian@uniklinik-freiburg.de // alexander.fabian@uksh.de

Registration

This study will be registered prior to enrollment of the first patient in the "Open Science Framework" (www.osf.io). Furthermore, the study will be registered on the German Clinical Trials Register (DRKS) and we will apply for a registration number of the ARO (*Arbeitsgemeinschaft Radiologische Onkologie*).

Financial Support

There is no external funding source or sponsor.

Acknowledgment

We acknowledge statistical counseling by Martin Treppner, Institute of Medical Biometry and Statistics, University Hospital Freiburg.

1 Introduction

Cancer patients face increased costs due to the disease or its treatment. Costs may be direct in terms of increased expenditures or indirect in terms of loss of income. This objective financial burden may cause subjective financial distress which in turn may result in financial toxicity (1). Financial toxicity has been linked to suboptimal treatment outcomes and patient satisfaction (2,3). Although financial toxicity has been studied extensively in the US, it has also been described in health care systems with public funding such as in Germany (4).

Radiotherapy is a major component in the treatment of cancer as approximately one out of two cancer patients receives radiotherapy at least once during the disease trajectory (5). Patients treated with radiotherapy face specific circumstances due to their treatment. This includes local side effects which may require topical supportive care which is often not reimbursed or also long treatment courses of up to 7 weeks. In this light, radiotherapy may cause both, increased direct costs as well as loss of income which may lead to financial toxicity. Yet only small exploratory studies have investigated financial toxicity in cancer patients treated with radiotherapy (Fabian et al. 2022, accepted, Strahlenther Oncol.). A better understanding of the prevalence and associated risk factors of financial toxicity is desirable to mitigate its potentially deleterious effect on treatment outcomes and patient satisfaction.

2 Methods

This is a cross-sectional, multi-center survey-based study. The questionnaire will be handed out to every patient meeting the eligibility criteria along with a study information. Informed consent is given if a patient returns the questionnaire in a sealed envelope. The survey is anonymous to ensure data protection.

Participating jDEGRO centers are (jDEGRO=junge DEGRO [Deutsche Gesellschaft für Radioonkologie]):

- University Hospital Freiburg, Department of Radiation Oncology, Freiburg
- University Hospital Schleswig-Holstein, Department of Radiation Oncology, Kiel
- University Hospital Cologne, Clinic and Policlinic for Radiation Oncology, Cyberknife and Radiotherapy, Cologne
- University Hospital Jena, Department of Radiotherapy and Radiation Oncology, Jena
- LMU University Hospital, Department of Radiotherapy and Radiation Oncology, Munich
- Additional German centers may follow and will be updated on the registration database DRKS

2.1 Eligibility criteria

Inclusion criteria are i.) cancer patient about to complete (+/- 2d) a course of radiotherapy, ii.) ability to understand and complete the questionnaire, iii.) >18 years, and iv.) patient gave informed consent.

The exclusion criterion is participation in the same study for another course of radiotherapy.

2.2 Outcomes and variables

All data will be retrieved from a questionnaire. The questionnaire was pilot tested on five voluntary, potentially eligible patients in March 2022. Thereafter, subtle changes to the layout were implemented. Furthermore, an additional sex category ("diverse") was added.

Outcome data originate from two questions on objective financial burden (additional costs and loss of income in the context of radiotherapy), one question on subjective financial distress (question 28 of the EORTC QLQ-C30), and a single question on patient satisfaction with radiotherapy (Numeric Rating Scale 0-10, modified based on (6)). At the conception of this study, there was no validated German measure to assess financial toxicity in terms of a single effect size. Therefore, we relied on subjective financial distress as surrogate parameter for financial toxicity as in a previous study (Fabian et al. 2022, accepted, Strahlenther Oncol.).

Variables include sociodemographic data (sex, age, marital status, education level, health insurance, exemption from copayments in social health insurance), data on the disease and radiotherapy (type of cancer, duration of radiotherapy, concomitant chemotherapy, out- vs. inpatient), data on occupation and finances (employment status, net household income), patient

distress (NRS distress thermometer (7)), and overall health-related quality of life (question 29 and 30 of the EORTC QLQ-C30).

2.3 Statistical analysis plan

One primary research question is the confirmation of the overall rate of financial toxicity per subjective financial burden (question 28 EORTC QLQ-C30) as detected in a previous exploratory study (Fabian et al. 2022, accepted, Strahlenther Oncol.). This study employed the same eligibility criteria. The questionnaire from this study was only slightly modified for the present study. Questions on outcomes or variables for the primary research questions were not modified. In the previous study, overall 31% of the patients reported subjective financial distress (Fabian et al. 2022, accepted, Strahlenther Oncol.).
To confirm this overall prevalence of financial subjective distress, we determined a sample size of n = 329 based on the expected prevalence of 31% and assuming a precision of the estimate of 5%. Accordingly, if the prevalence of subjective financial distress falls into the 95% binomial exact confidence interval of 26.04-36.31%, this primary research question will be regarded as confirmed. The subgrades of subjective financial distress were rated by patients as 21% “a little”, 6% “quite a bit”, and 4% “very much” in the previous study. Yet of note, we considered the endpoint to be binary (absence/presence of financial distress) to determine the necessary sample size for estimating the prevalence.

Another primary research question is the confirmation of risk factors of financial toxicity per subjective financial distress as shown in the previous study (Fabian et al. 2022, accepted, Strahlenther Oncol.). Statistically significant risk factors included lower net household income (OR= 0.28; p = 0.01), higher direct costs (OR= 2.78; p = 0.021), and higher loss of income (OR= 2.48 p = 0.025) per multivariate ordinal regression. Active employment status (OR= 16.96; p = 0.067) and lower health-related quality of life (OR= 0.51; p = 0.058) were also associated with higher subjective financial distress per multivariate ordinal regression.
To be able to confirm these risk factors, we conducted a sample size calculation based on a logistic regression model using G*Power Version 3.1.9.6 (Faul et al. 2007,2009) since sample size determination for ordinal regression models requires potentially complex simulation approaches (8,9). We used a conservatory approach by calculating the sample size for the smallest response subcategory of the dependent variable (=”very much” subjective financial distress), which was reported by 4% of the patients based on the previous study as mentioned above. We conducted the sample size calculation based on a type I error of $\alpha = 0.05$, a power of 0.8, an odds ratio for the expected effect size of 0.5, and a correction factor for covariates (for details, see G * Power 3.1 manual, p. 70, ‘R^2 other X’), the resulting sample size was n = 504 patients for a two-sided z-test. If the risk factors mentioned above significantly predict the extent of subjective financial distress per ordinal regression analysis, these risk factors will then be regarded as confirmed in the setting of our study.

Secondary research questions include the exploration of additional variables as risk factors for financial toxicity (e.g. distress, duration of radiotherapy) as well as the exploration of an association of subjective financial distress and patient satisfaction.

Participation rate will be described based on returned questionnaires and the number of cancer patients that finished a course of radiotherapy during the study period. This approach underestimates the participation rate as also potentially ineligible patients (e.g. unable to understand questionnaire) will be included in the latter number. However, this approach appears reasonably feasible due to the large overall patient numbers in each center.

2.4 Duration of the study

To achieve the largest planned sample size of 504 patients we plan patient accrual during two consecutive months. This assumption is based on five participating centers, 2.000 treated patients per year per center, and a participation rate of 30% resulting in 600 patients after 60 days of accrual. The participation rate of 30% is a conservatory estimate based on the previous study (53.3%) and based on the underestimating definition of participation rate (as mentioned in chapter 2.3).
In case the planned sample size will be achieved earlier, accrual will continue to complete two months of accrual in order to facilitate additional exploratory analyses. In case the planned sample size should not be achieved after two months, accrual will continue until the planned sample size will be achieved if feasible in a reasonable amount of time. All centers will report the number of included patients on a weekly basis to the study coordinators.

3 Ethical aspects and data protection

3.1 Ethical and legal issues

We do not expect any ethical or legal issues in the context of this study due to the non-interventional, cross-sectional, and anonymous study design. Study participation is voluntary. Participation or non-participation will not influence a patients' care by any means. Patient insurance is not necessary for the purpose of this study as no intervention other than the questionnaire administration at a routine visit will be administered.

The Ethics Committee of the Medical Faculty of Christian-Albrecht-University of Kiel will be asked for approval of the study prior to enrolment of the first patient. Each participating center will apply for approval of the respective Ethics Committee prior to enrolment of the first patient at their site.

3.2 Data protection and data handling

We do not expect any issues concerning data protection as all data retrieved within the study is anonymous due to the large number of potentially eligible patients (approx. over 2.000 patients treated in each radiotherapy department per year). There will not be any form of pseudonymization. Therefore, it will not be possible to link data to an individual patient.

As described above, the questionnaire and a patient information sheet will be handed out to eligible patients. Therefore, informed consent is given if a patient returns the questionnaire in a sealed envelope to the hospital staff. At the end of the study, the pooled envelopes will be opened only by members of the local study team. The anonymous data arising from the questionnaires will be extracted into an Excel file in each participating center. The Excel file will be protected by a password given by the local study team. Participating centers will send the Excel file via mail to the study coordinators. The passwords will be transmitted via phone call. The data of all centers will be pooled and analyzed by the study coordinators.

Due to the anonymous character of data in this study, the EU General Data Protection Regulation/ *Datenschutz-Grundverordnung* (GDPR / *DSGVO*) does not apply per Recital 26 GDPR / *Erw.Gr. 26 DSGVO*.

4 Publication

We seek publication of results of this study. A priori, each participating center is respected for co-authorship (1 co-author) if at least 50 patients have been recruited in the respective center. Additional co-authors may be respected per authorship guideline of the jDEGRO if feasible.

5 References

1. Witte J, Mehlis K, Surmann B, Lingnau R, Damm O, Greiner W, u. a. Methods for measuring financial toxicity after cancer diagnosis and treatment: a systematic review and its implications. Annals of Oncology. 2019;30(7):1061–70.

2. Yabroff KR, Bradley C, Shih YCT. Understanding Financial Hardship Among Cancer Survivors in the United States: Strategies for Prevention and Mitigation. Journal of Clinical Oncology [Internet]. 2019 [zitiert 1. November 2021]; Verfügbar unter: https://ascopubs.org/doi/pdf/10.1200/JCO.19.01564

3. Perrone F, Jommi C, Maio MD, Gimigliano A, Gridelli C, Pignata S, u. a. The association of financial difficulties with clinical outcomes in cancer patients: secondary analysis of 16 academic prospective clinical trials conducted in Italy. Annals of Oncology. 2016;27(12):2224–9.

4. Longo CJ, Fitch MI, Banfield L, Hanly P, Yabroff KR, Sharp L. Financial toxicity associated with a cancer diagnosis in publicly funded healthcare countries: a systematic review. Supportive Care in Cancer. Oktober 2020;28(10):4645–65.

5. Lievens Y, Borras JM, Grau C. Provision and use of radiotherapy in Europe. Molecular Oncology. 2020;14(7):1461–9.

6. Gomez-Cano M, Lyratzopoulos G, Abel GA. Patient Experience Drivers of Overall Satisfaction With Care in Cancer Patients: Evidence From Responders to the English Cancer Patient Experience Survey. Journal of Patient Experience. 1. Oktober 2020;7(5):758–65.

7. Mehnert-Theuerkauf A, Müller D, Lehmann C, Koch U. Die deutsche Version des NCCN Distress-Thermometers: Empirische Prüfung eines Screening-Instruments zur Erfassung psychosozialer Belastung bei Krebspatienten. Zeitschrift für Psychiatrie Psychologie und Psychotherapie. 1. Januar 2006;54:213–23.

8. Faul F, Erdfelder E, Lang A-G, Buchner A. G*Power 3: A flexible statistical power analysis program for the social, behavioral, and biomedical sciences. Behavior Research Methods. 1. Mai 2007;39(2):175–91.

9. Faul F, Erdfelder E, Buchner A, Lang A-G. Statistical power analyses using G*Power 3.1: Tests for correlation and regression analyses. Behavior Research Methods. 1. November 2009;41(4):1149–60.

Signature

22.03.2022 ______________________

Study coordinator Dr. Alexander Fabian, MBA
