# Supplementary material for: Psychosocial distress in cancer patients undergoing radiotherapy: a prospective national cohort of 1042 patients in Germany
Source: J Cancer Res Clin Oncol. 2023 May 10;149(11):9017–24. doi: 10.1007/s00432-023-04837-5 (PMC10374761; doi:10.1007/s00432-023-04837-5)
Supplement: Supplementary file 2 — Supplementary file2 (PDF 125 KB) [file 432_2023_4837_MOESM2_ESM.pdf]

English translation – see below for the original version in German

Patient survey on financial burden in the context of radiotherapy

Socio-demographic data

1.) Sex: ☐ female ☐ male ☐ diverse

2.) How old are you? ____________ years

3.) How do you live?

☐ alone ☐ with partner

4.) Your highest school graduation:

☐ None ☐ < 10 years of education *(„Hauptschulabschluss")* ☐ 10 years of education *(„Realschulabschluss")* ☐ > 10 years of education *(„Abitur")*

5.) What is your health insurance? ☐ social ☐ private

6a.) if social: Are you exempt from copayments? ☐ Yes ☐ No

Clinical data and therapy

6.) For which type of cancer are you currently treated with radiotherapy?

☐ Breast cancer ☐ Prostate cancer ☐ Head and Neck cancer
☐ Lung cancer ☐ Esophageal cancer ☐ Rectal Cancer
☐ other: *(please note)*

____

7.) How long does your current treatment with radiotherapy take? ______ days
(only duration of treatment including weekends but not including previous computer tomography or ambulatory visits prior to treatment)

8.) Did you receive chemotherapy along with your radiotherapy?

☐ Yes ☐ No *(hormone therapy excluded)*

9.) Have you been hospitalized during your radiotherapy?

☐ Yes, throughout ☐ Yes, in part ☐ No, not at all

10.) Overall, how would you rate your radiotherapy care?
Please mark the respective number.

1 2 3 4 5 6 7 8 9 10

not satisfied at all *very much satisfied*

Employment and financial data

11.) What is your current employment status?

☐ Employed ☐ Self-employed ☐ Official ☐ Unemployed ☐ Retired

12.) What is your monthly net household income? This means, the money at your disposal after deduction of taxes and social insurance contributions.

☐ < 1.300 € ☐ 1.301 – 1.700 € ☐ 1.701 – 2.600 €
☐ 2.601 – 3.600 € ☐ 3.600 – 5.000 € ☐ > 5.000 €

13.) Did you have any loss of income due to your radiotherapy?

☐ Yes ☐ No

13a.) If yes, to which degree overall?

☐ < 100 € ☐ 101 - 500€ ☐501 - 1500€ ☐ 1501 - 2500€ ☐ > 2500€

14.) Did you have any additional costs due to your radiotherapy? (e.g. copayments, medicines exempt from reimbursement etc.)? ☐ Yes ☐ No

14a.) If yes, to which degree overall?

☐ < 100 € ☐ 101 - 500€ ☐501 - 1500€ ☐ 1501 - 2500€ ☐ > 2500€

14b.) What were the reasons for additional costs? (*multiple choices possible*)

☐ Copayments (e.g. drugs, therapeutic products, medical aids, hospital stay etc.)
☐ Transportation
☐ Drugs/Products for supportive care to alleviate side effects of radiotherapy that are exempt from reimbursement (e.g. care lotions or washes)
☐ Other (e.g. PET-CT) *(please note)*:

15.) *During the past week:* has your physical condition or medical treatment caused you financial difficulties?

☐ Not at all ☐ A little ☐ Quite a bit ☐ Very much

Distress

16.) Please cross the number (0-10) that best describes how much distress you have been experiencing in the past week including today.

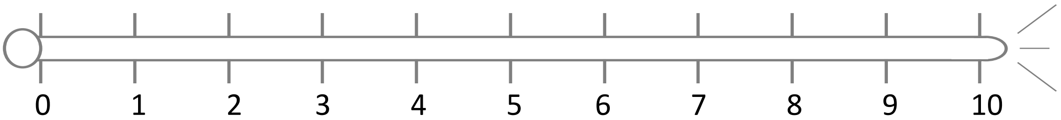

0 1 2 3 4 5 6 7 8 9 10

no distress *extreme distress*

Health status and quality of life

For the following questions please circle the number between 1 and 7 that best applies to you:

17.) How would you rate your overall health during the past week?

1 2 3 4 5 6 7

Very poor *Excellent*

18.) How would you rate your overall quality of life during the past week?

1 2 3 4 5 6 7

Very poor *Excellent*

<u>Patientenfragebogen zur finanziellen Belastung im Rahmen der Strahlentherapie</u>

Sozio-Demographische Aspekte

1.) Geschlecht: ☐ weiblich ☐ männlich ☐ divers

2.) Wie alt sind Sie? ____________ Jahre

3.) Wie leben Sie? ☐ alleine ☐ mit Partner

4.) Ihr höchster Schulabschluss:

☐ kein Abschluss ☐ Hauptschulabschluss ☐ Realschulabschluss ☐ Abitur

5.) Wie sind Sie krankenversichert? ☐ gesetzlich ☐ privat

5a.) falls gesetzlich: Sind Sie von Zuzahlungen befreit? ☐ Ja ☐ Nein

Angaben zu Erkrankung und Therapie

6.) Für welche Tumorerkrankung erhalten Sie aktuell die Strahlentherapie?

☐ Brustkrebs ☐ Prostatakrebs ☐ Rachen-/Mundhöhlen-/ Kehlkopfkrebs
☐ Lungenkrebs ☐ Speiseröhrenkrebs ☐ Darmkrebs
☐ sonstige, nicht aufgeführte Tumorerkrankung (*bitte notieren*):

____

7.) Wie lange dauert(e) ihre aktuelle Behandlung in der Strahlentherapie? ______ Tage (*nur Behandlungszeitraum einschließlich der Wochenenden, jedoch nicht voriges Planungs-CT oder Arztgespräch*)

8.) Haben Sie parallel zur aktuellen Strahlentherapie eine Chemotherapie erhalten?

☐ Ja ☐ Nein *(Hormontherapie zählt nicht dazu)*

9.) Erfolgte die Strahlentherapie stationär, d.h. waren Sie stationär im Krankenhaus aufgenommen? ☐ Ja, durchgehend ☐ Ja, teilweise ☐ Nein, gar nicht

10.) Wie zufrieden sind Sie insgesamt mit Ihrer Behandlung in der Strahlentherapie? Bitte kreuzen Sie die für Sie zutreffende Zahl an.

1 2 3 4 5 6 7 8 9 10

gar nicht zufrieden *sehr zufrieden*

Berufliche und finanzielle Aspekte

11.) Wie ist Ihr aktueller Erwerbsstatus?

☐ Angestellt ☐ Selbstständig ☐ Beamter/in ☐ nicht erwerbstätig ☐ berentet

12.) Wie hoch ist ihr gesamtes <u>monatlich verfügbares Haushalts-Nettoeinkommen</u>, also das Geld, das nach Abzug der Steuern und Sozialversicherungsbeiträge dem gesamten Haushalt übrigbleibt?

☐ unter 1.300 € ☐ 1.301 – 1.700 € ☐ 1.701 – 2.600 €
☐ 2.601 – 3.600 € ☐ 3.600 – 5.000 € ☐ über 5.000 €

<u>Bitte wenden</u> **<u>Rückseite ➜</u>**

13.) Sind Ihnen in Folge Ihrer aktuellen Behandlung in der Strahlentherapie Einkommenseinbußen entstanden? ☐ Ja ☐ Nein

13a.) Wenn ja, in welcher Höhe insgesamt?

☐ unter 100 € ☐ 101 - 500€ ☐501 - 1500€ ☐ 1501 - 2500€ ☐ über 2500€

14.) Haben Sie in Folge Ihrer aktuellen Behandlung in der Strahlentherapie höhere Ausgaben (z.B. Zuzahlungen, nicht-erstattete Arzneien etc.)? ☐ Ja ☐ Nein

14a.) Wenn ja, in welcher Höhe insgesamt?

☐ unter 100 € ☐ 101 - 500€ ☐501 - 1500€ ☐ 1501 - 2500€ ☐ über 2500€

14b.) Wodurch fallen Ihnen höhere Ausgaben an? (*Mehrfachnennungen möglich*)

☐ Zuzahlung (z.B. Arznei- / Heil- / Hilfsmittel, Krankenhausaufenthalt etc.)
☐ Fahrtkosten
☐ Arzneimittel/Pflegeprodukte zur Linderung von Nebenwirkungen der Strahlentherapie, die nicht erstattet werden (z.B. pflegende Salben/Spülungen)
☐ Sonstiges (z.B. PET-CT) *(bitte notieren)*:

____

15.) *Während der letzten Woche:* Hat Ihr körperlicher Zustand oder Ihre medizinische Behandlung für Sie finanzielle Schwierigkeiten mit sich gebracht?

☐ überhaupt nicht ☐ wenig ☐ mäßig ☐ sehr

Belastung

16.) Bitte kreuzen Sie die Zahl (0-10) an, die am besten beschreibt, wie belastet Sie sich in der letzten Woche einschließlich heute gefühlt haben.

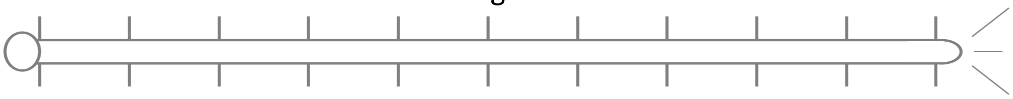

0 1 2 3 4 5 6 7 8 9 10

gar nicht belastet *extrem belastet*

Gesundheit und Lebensqualität

Bitte kreuzen Sie bei beiden folgenden Fragen die Zahl zwischen 1 und 7 an, die am besten auf Sie zutrifft:

17.) Wie würden Sie insgesamt Ihren Gesundheitszustand während der letzten Woche einschätzen?

1 2 3 4 5 6 7

sehr schlecht *ausgezeichnet*

18.) Wie würden Sie insgesamt Ihre Lebensqualität während der letzten Woche einschätzen?

1 2 3 4 5 6 7

sehr schlecht *ausgezeichnet*

Vielen Dank für Ihre Zeit und Teilnahme

Bitte geben Sie den Fragebogen im verschlossenen Umschlag beim Klinikpersonal ab.
