# Supplementary material for: Psychosocial distress in cancer patients undergoing radiotherapy: a prospective national cohort of 1042 patients in Germany
Source: J Cancer Res Clin Oncol. 2023 May 10;149(11):9017–24. doi: 10.1007/s00432-023-04837-5 (PMC10374761; doi:10.1007/s00432-023-04837-5)
Supplement: Supplementary file 3 — Supplementary file3 (PDF 55 KB) [file 432_2023_4837_MOESM3_ESM.pdf]

Psychosocial distress in cancer patients undergoing radiotherapy – a prospective national cohort of 1042 patients in Germany

Journal of Cancer Research and Clinical Oncology

Fabian, A.[1] et al,
[1] Department of Radiation Oncology, University Hospital Schleswig-Holstein, 24105 Kiel, Germany, alexander.fabian(at)uksh.de

Supplementary Table 1 Additional costs or loss of income due to radiotherapy among those patients who answered the question on distress (n = 1042). Numbers may not add up to 100% due to rounding error or missing values.

Additional costs due to radiotherapy	None	32% (338)
	Yes, but degree not reported	12% (123)
	< 100 €	14% (142)
	101 – 500 €	29% (306)
	501 – 1.500 €	8% (78)
	1.501 – 2.500 €	1% (13)
	> 2.500 €	1% (9)
Loss of income due to radiotherapy	None	70% (732)
	Yes, but degree not reported	2% (24)
	< 100 €	2% (24)
	101 – 500 €	13% (131)
	501 – 1.500 €	7% (72)
	1.501 – 2.500 €	2% (21)
	> 2.500 €	1% (15)
