# Supplementary material for: Psychosocial distress in cancer patients undergoing radiotherapy: a prospective national cohort of 1042 patients in Germany
Source: J Cancer Res Clin Oncol. 2023 May 10;149(11):9017–24. doi: 10.1007/s00432-023-04837-5 (PMC10374761; doi:10.1007/s00432-023-04837-5)
Supplement: Supplementary file 4 — Supplementary file4 (PDF 65 KB) [file 432_2023_4837_MOESM4_ESM.pdf]

Psychosocial distress in cancer patients undergoing radiotherapy – a prospective national cohort of 1042 patients in Germany

Journal of Cancer Research and Clinical Oncology

Fabian, A.[1] et al,
[1] Department of Radiation Oncology, University Hospital Schleswig-Holstein, 24105 Kiel, Germany, alexander.fabian(at)uksh.de

Supplementary Table 2 Summary parameters of the multiple regression model

**Model Summary**	
Number of observations	723
F(12, 710)	34.8
Prob > F	<0.001
R-squared	0.370
Adj. R-squared	0.359
Durbin-Watson-Statistics	2.006
Assumption of homoscedasticity	Not violated per balanced distribution of residuals around baseline
Assumption of normality and linearity	Not violated per Q-Q plot
Variable entry method	Simultaneous entry
